# Integrative Taxonomy for Continental-Scale Terrestrial Insect Observations

**DOI:** 10.1371/journal.pone.0037528

**Published:** 2012-05-29

**Authors:** Cara M. Gibson, Rebecca H. Kao, Kali K. Blevins, Patrick D. Travers

**Affiliations:** The National Ecological Observatory Network, Boulder, Colorado, United States of America; Biodiversity Insitute of Ontario - University of Guelph, Canada

## Abstract

Although 21^st^ century ecology uses unprecedented technology at the largest spatio-temporal scales in history, the data remain reliant on sound taxonomic practices that derive from 18^th^ century science. The importance of accurate species identifications has been assessed repeatedly and in instances where inappropriate assignments have been made there have been costly consequences. The National Ecological Observatory Network (NEON) will use a standardized system based upon an integrative taxonomic foundation to conduct observations of the focal terrestrial insect taxa, ground beetles and mosquitoes, at the continental scale for a 30 year monitoring program. The use of molecular data for continental-scale, multi-decadal research conducted by a geographically widely distributed set of researchers has not been evaluated until this point. The current paper addresses the development of a reference library for verifying species identifications at NEON and the key ways in which this resource will enhance a variety of user communities.

## Introduction

Wilson [1] estimated that there are conservatively 10 million species on our planet yet to be described. With fewer than 6,000 systematists, each would need to describe nearly 2,000 novel species in his or her lifetime. Given the six months per species that this activity requires (also conservative), it would take 1,000 years for each systematist to completely describe Earth’s species. Systematists or professional taxonomists’ work of novel species descriptions or ascribing species to particular groups requires years of specialized training and credit in this discipline is primarily for these activities. The species “ID services” so needed by epidemiologists, pest managers, agriculturalists and ecologists, to name a few, also require systematist’s great skill, however, taxonomists typically face criticism for not rendering these ID services more rapidly. Some researchers who require specimen identifications then circumvent the use of expert taxonomists by relying on parataxonomists (akin to a paralegal or a paramedic), or solely on molecular data. Unfortunately, shortcuts often result in inaccurate species identifications and have caused incredibly costly errors that spread insidiously through time [2], [3]. Locke and Coates [4], for instance, conservatively estimated that inappropriate taxonomic practices for a single Caribbean coral species cost just under 4 million USD through misallocation of researcher time. Integration with professional taxonomists who are the experts for particular groups is essential for constructing a reliable and effective specimen identification reference library.

NEON is a national-scale research platform for analyzing and understanding the impacts of climate, land-use, and invasive species on ecology. Using standardized protocols and an open data policy, NEON will feature field observations, sensor networks and experiments, and advanced cyberinfrastructure to record and archive ecological data for 30 years across the continental US, Alaska, Hawai’i and Puerto Rico (http://www.neoninc.org/science/domains [5]). During observatory operations field observations and analyses of microbes, plants, ground beetles, mosquitoes, birds, and small mammals will prove data on biodiversity, population dynamics, productivity, phenology, infectious disease, and biogeochemistry. (See the NEON Science Strategy document: http://www.neoninc.org/science/sciencestrategy).

A NEON design committee [6] selected ground beetles (Coleoptera: Carabidae) as a focal taxon as they are distributed over the extent of the observatory, straightforward to sample, relatively well known taxonomically (∼3,000 species in NEON extent), influence trophic structure, and have been used in a variety of other contexts as indicators for change [7], [8], [9]. Mosquitoes (Diptera: Culicidae) were selected as they are distributed over the observatory’s extent, straightforward to sample, relatively well known taxonomically (∼200 species in NEON extent), important disease vectors and sensitive to environmental change [10], [11]. In the US, wild insects provide 57 billion USD per year for the four ecosystem services of dung burial, pest control, pollination, and wildlife nutrition [12]. Clearly, understanding the ecology of US insects has a significant financial impact in addition to its implicit biological importance.

During observatory operations NEON will collect large numbers of specimens of common species which must be identified and reported to the community with confidence estimates (i.e. in the same way that NEON instrument measurements will be reported). As such, members of the taxonomic community recommended molecular data to ameliorate the taxonomic needs of NEON at a National Museum of Natural History (NMNH)-NEON Workshop on the Curation of Biological Specimens in 2008. Specifically, their recommendation was for the use of 658 bp of the mitochondrial cytochrome c oxidase subunit 1 (CO1) gene that is used by a global community for identifying animals. The use of this gene marker for species identifications is known as DNA barcoding (for recent reviews see [13], [14], [15]). NEON plans to use CO1 initially, and supplementary genes if required, as an additional method for the terrestrial insect identifications by first building integrated reference libraries that include morphological, behavioral, phenological, ecological and distribution data in addition to sequence data. The creation of this library requires specimens with accurate identifications using either freshly collected material or curated specimens in collections. In observatory operations, molecular sequences, in addition to expert taxonomists’ identifications, will be used as a separate approach for a subset of the specimens that will be morphologically identified by parataxonomists. DNA barcoding will facilitate rapid verifications of common specimens that are of limited interest to, and would in fact impede, the professional systematics community. Additionally, we anticipate that the molecular data will unveil novel taxa that will be of interest to taxonomists.

In 2009, NEON began the development of a specimen identification library for ground beetles and mosquitoes. The currently established DNA barcoding methods were evaluated for their efficacy, and the first site-specific sequences, species lists and appropriate workflows for the high throughput of specimens were developed. The work presented here is an introduction to the first 738 Barcode of Life Datasystem (BOLD) barcode compliant CO1 sequences (those records with formal barcode status as defined by Ratnasingham and Hebert [16]) and that NEON has identified as reference sequences (see definition below) and 630 additional records produced by this work that are not reference quality at this time. The publicly available reference records will be an appropriate method for integrating the terrestrial invertebrate observations with others made by the observatory and the wider scientific community. The wealth of specimens, DNA and associated metadata that will be gathered by NEON for this effort will represent a major resource for the ecological, systematic, medical entomological, and citizen science communities.

## Materials and Methods

All field collections were made in 2008, 2009 and 2010 (see Table 1) at NEON candidate sites. Field collections were made at the Central Plains Experimental Range (CPER), CO (2008, 2009), Sterling Agricultural Field, CO (2009), Niwot Ridge, CO (2009), Fraser Experimental Forest, CO (2009), and Harvard Forest, MA (2010). All field necessary permits were obtained from the following people and organizations in respective order to the field sites listed above: Mary Ashby, U.S. Department of Agriculture, Agriculture Research Service; Gilbert Lindstrom, private owner; William Bowman, University of Colorado at Boulder, U.S. Forest Service; Kelly Elder, U.S. Forest Service; and Edythe Ellin, Harvard University. Ground beetles were collected in pitfall traps (2 nested plastic bowls, ∼15 cm deep, 13 cm diameter, with cover 2.5 cm above bowl) set flush to ground level. Each trap was filled with propylene glycol:water (2∶1). Traps were checked and reset once per week. Upon first collection from the field the 2008 material was stored in 75% ethanol and identified by researchers. The 2009 and 2010 insects were rinsed with water, then rinsed with 95% ethanol and then transferred to 95% ethanol. The ethanol was then changed before final storage in 95% ethanol at −20**°**C. Beetles from the family Carabidae (hind coxae separating the first abdominal segment and 5-5-5 tarsal formula) were sorted from the samples, pinned, labeled, identified to morphospecies and then sent to taxonomists for expert morphological identification (Dr. Foster Purrington, Ohio State Univ., Dr. Wendy Moore, Jason Schaller, Univ. of Arizona, in 2009, and Moore and Schaller, in 2010). If there were more than 20 of the same morphospecies from a pitfall trap, the first 20 were pinned or pointed and the remainder were counted and stored in 95% ethanol. The rest of the trap samples (termed “bycatch”) were stored in 95% ethanol. Of the 479 specimens in 2008, the 1,575 specimens collected in 2009 and the 250 collected in 2010, representative sub-samples (554 specimens) were pinned, labeled and prepared for DNA extraction and sequencing (with duplication of 21 specimens to verify sequencing facility efforts). All beetle specimens and associated genomic extracts from these prototype efforts are housed at NEON headquarters in Boulder, CO.

**Table 1:** Numbers of NEON sub-sampled ground beetle and mosquito specimens sequenced from both field and museum campaigns.

Sampling Locations & Dates		NEONT		NEONZ		% Success
		B	M	B	M	
F	Central Plains Experimental Range, CO B: 9/10-10/17/08	2	NA	51	NA	4
F	Central Plains Experimental Range, CO B: 7/14-8/04/2009;M: 7/14-9/1/09	33	29	18	9	70
F	Sterling Agricultural Field, CO B: 8/17-8/31/2009;M: 8/14-8/26/09	76	22	14	4	84
F	Niwot Ridge, CO B: 7/23-8/06/09; M: 7/14-8/18/09	17	14	11	5	66
F	Fraser Experimental Forest, CO B: 7/22-8/12/09;M: 7/13-8/19/09	37	58	15	33	66
F	Harvard Forest, MA B & M: 6/07-7/28/10	171	122	88	16	74
F	Total	336	245	197	67	69
A	University of Colorado Museum of Natural History, CO10/09	4	NA	36	NA	10
A	National Museum of Natural History, DC 10/10	6	NA	31	NA	16
A	Walter Reed Biosystematics Unit, MD 02/11	NA	60	NA	139	30
A	C.P. Gillette Museum of Natural History, CO 12/10 &05/11	87	NA	152	NA	36
A	Total	97	60	219	139	30
F + A	Total	433	305	416	206	54

Ground beetle  =  B and mosquito  =  M specimens from field  =  F and museum archive  =  A efforts. NA  =  not applicable. Sites and specific sampling dates are listed with % success calculated by the record joining the reference library (the ‘NEONT’ project in BOLD) divided by all sequenced specimens; this includes those records requiring more information (the ‘NEONZ’ project). The records in ‘NEONZ’ are public and have been of utility to our research and we anticipate for others’ also (see text).

Mosquitoes were collected using CO_2_-baited Center for Disease Control (CDC) light traps (John W. Hock, FL) in 2009 and using CO_2_-baited CDC light traps, gravid traps (John W. Hock, FL) and BG sentinel traps (BioQuip, CA) in 2010. Traps were deployed from dusk until dawn two nights per week. Mosquitoes were sorted from the samples by field technicians and identified morphologically by taxonomists (led by Dr. Michael Weissmann) at Colorado Mosquito Control (Brighton, CO) and a subset of the 2010 specimens by Dr. Richard Darsie, Jr. retired, Univ. of Florida's Medical Entomology Laboratory. Of the 1,438 specimens collected in 2009 and the 4,194 specimens collected in 2010, representative sub-samples (321 specimens) were pinned, labeled and prepared for DNA extraction and sequencing (with duplication of 9 specimens to verify sequencing facility efforts). The remainder were stored at −20°C sorted by trap, date and species. All mosquito specimens and associated genomic extracts from these prototype efforts are housed at NEON headquarters in Boulder, CO.

In addition to the field collections, five museum trips were conducted which resulted in the sub-sampling of 517 specimens (see Table 1). Over the course of our initial museum archive visits, we developed criteria for the selection of specimens. We worked with a single drawer at a time so that specimens were returned to their appropriate locations and the risk of damage minimized. Specimens that were collected from 1965 to present were considered first, with more recently collected specimens being selected preferentially. We prioritized specimens with clear locality data and known species determiners in lieu of unknowns. Long series of specimens were preferred and three specimens of each species were selected with the widest geographic range possible (though specimens of the same species were typically from the same lot). For ground beetle specimens, males were prioritized ahead of females due to their greater ease of morphological identification.

One leg from each specimen was removed and placed into a 96 well plate with a leg priority of right then left midleg, right then left hindleg, right then left foreleg. Microwell plates were then sent to the Smithsonian Laboratories for Analytical Biology (Silver Spring, MD; 2009), Pisces Molecular (Boulder, CO) or the Biodiversity Institute of Ontario for genomic extraction and sequencing (Guelph, ON; 2010 and 2011). Polymerase Chain Reaction amplification of the CO1 gene was carried out using the general invertebrate CO1 primers and methods following Folmer et al. [17] to generate bi-directional reads (see http://www.ccdb.ca/pa/ge/research/protocols for additional extraction and sequencing details). NEON staff checked reads against both the BOLD database and GenBank to verify sequence identities. All records from this work have been uploaded to BOLD and are public. ‘NEONT’ records represent the following: singletons with no (currently) conflicting data, monophyletic groups with two or more specimens of the same name, paraphyletic groups with two or more specimens of the same name in each clade. BOLD and GenBank are used for guidance, however, not definitively, as the source of identification is often not available nor how carefully sequence reads were checked. Further, some of the records visible in the BOLD ID engine are private and cannot be properly accessed or evaluated. ‘NEONZ’ represents those records that are either not barcode compliant or require additional information or expertise to resolve them to the level where they would represent reliable reference records. NEON has a yearly management plan to analyze all of the ‘NEONT’ and ‘NEONZ’ records in BOLD and update them as new information pertinent to these records becomes available.

## Results

An initial 1,404 tissue samples were taken from ground beetles and mosquitoes from both field material and vouchered material held in collections. Initial amplification success was 83% from material collected on average 7.6 years (median  = 1 year) prior to the sequencing attempt. From these, 738 barcode compliant reference sequences were recovered and entered into ‘NEONT’. Another 630 records, entered in the project ‘NEONZ’, could become reference records with additional information, save for 8 specimens that are neither ground beetles nor mosquitoes.

For the barcode compliant ground beetle sequences in ‘NEONT’, there were 433 specimens from 140 species, 47 genera and 6 subfamilies. The intraspecific distances calculated using Kimura 2 Parameter model (from the BOLD analysis tools) were a maximum of 2.34% and minimum 0%. The distances to the next nearest neighbors were a maximum of 15.99% and minimum 0%. There were 87 singletons and 29 species with five or more sequences. Twenty of these species exhibited below 1% maximum intraspecific distance, and 8 of these species exhibited between 1 and 2% maximum intraspecific distance. The remaining species, *Amara alpina*, exhibited a maximum intraspecific distance of 2.02%. Regarding the relationship of the ages of the ground beetle specimens to sequencing success, 66% of the compliant beetle specimens collected fewer than 2 years previous were successful (378/572 submitted), specimens between 2 and 25 years were 24% successful (49/203) and specimens greater than 25 years old were 11% successful (6/62, 2 specimens had no collection date but were assumed to be in this category). At the time of this writing (November 2011), NEON had contributed 4% of the total North American ground beetle records to BOLD.

For the barcode compliant mosquito sequences in ‘NEONT’, there were 305 specimens from 62 species, 8 genera and 2 subfamilies. The intraspecific distances were a maximum of 10.88% and minimum 0%. The distances to the next nearest neighbors were a maximum of 14.72% and minimum 0.15%. There were 18 singletons and 21 species with five or more sequences. Six of these species exhibited below 1% maximum intraspecific distance, and 9 of these species exhibited between 1 and 2% maximum intraspecific distance. The remaining 6 species exhibited maximum intraspecific distances as follows, *Aedes communis* 2.03%, *Aedes trivittatus* 2.18%, *Aedes aurifer* 2.66%, *Aedes hexodontus* 6.1%, *Coquillettidia perturbans* 8.25% and *Aedes fitchii* 10.02%. Regarding the relationship of the ages of the mosquito specimens to sequencing success, specimens collected fewer than 2 years previous were 79% successful (245/312), specimens between 2 and 25 years were 100% successful (4/4) and specimens greater than 25 years old were 31% successful (25/81). There were many specimens with no collection date (likely older than 25 years) and these were 27% successful (31/114). At the time of this writing, NEON had contributed 29% of the total North American mosquito records to BOLD.

We also evaluated two novice parataxonomists in 2009 and 2010 for their ability to parse specimens into groupings that were consistent with expert taxonomists. These technicians sorted and pinned the 1,575 ground beetle specimens from 2009. A representative subset of each morphospecies was then sent for professional taxonomic morphological identification and DNA sequencing. In one such shipment of 673 specimens, the parataxonomists identified 37 unique morphospecies, which the experts identified as 35 unique species (94% success; 35 unique groups properly identified and two inappropriately split). The same technicians sorted and prepared the 2010 beetle specimens. From their previous years’ experience, and with a small teaching collection that was developed from NEON specimens, the parataxonomists were able to identify genus correctly for 48% of these species, tribe for 63%, and subfamily for 74% of specimens. This higher resolution sorting effort expedited the experts’ workload upon receiving the specimens. The parataxonomists identified 26 unique morphospecies from the 250 specimens, which the experts identified as 27 unique species (96% success; 26 unique groups properly identified and one group inappropriately lumped).

## Discussion

This research is focused on the prototype evaluation of 658 bp of CO1, known as a DNA barcode, to validate species identifications of terrestrial invertebrate specimens collected by NEON. NEON has divided its efforts into two public projects at this phase. Entries in the ‘NEONT’ project are records that are well supported (or not negated) by other data. Entries in the ‘NEONZ’ project are either not barcode compliant or show a discordance between the morphological and molecular identifications when compared with other NEON specimens (collected during the same sampling bout) or morphologically identified species whose sequence data were not consistent with existing DNA barcode records in BOLD or GenBank. For some of the entries in the ‘NEONZ’ project (obvious contaminants, etc.), the confounding sequences have been removed from the record so that they are not part of the BOLD species identification engine.

In general, this approach has been successful, 83% initial amplification success and 69% and 30% success in DNA barcode reference record creation from field collected and museum sub-sampled material respectively (see Table 1). As NEON is taking a site-based approach during the full operations field sampling, we feel certain that the DNA barcodes will serve as a powerful additional line of inquiry into understanding species diversity at local scales given that the majority of sequenced specimens with at least 5 replicate conspecifics exhibited less than 2% sequence divergence. However, there have been some important lessons learned as well as standing issues uncovered. For instance, we suspect that the 75% ethanol concentration for the 2008 CPER field samples dramatically lowered the amplification success of this fresh, field-collected material (4% relative to >66% for other NEON field campaign samples). A minimum ethanol concentration of 95% is necessary for optimal DNA preservation (Lee Weigt pers. comm.). Further, some taxa collected from the same locality exhibited a great deal of intraspecific variation, e.g. *Aedes fitchii* mosquitoes from Fraser Experimental Forest in Grand County, CO showed 5.54% variation. This particular species has been shown to exhibit great intraspecific variation previously, and further there is evidence that *Ae. grossbecki* may hybridize with *Ae. fitchii* [18]. From NEON’s point of view, specimens collected subsequently by NEON should cluster within one of the clades currently recovered from specimens in the reference library.

During the construction of the observatory, additional DNA barcode records will be created through both field campaigns and museum visits with an emphasis on the latter. Despite the relatively lower sequencing success (see Table 1, field  = 69% and museum  = 30%), records from museum archives are more efficient when compared to the costs of managing field campaigns (equipment and technicians) and shipping specimens to taxonomists. Further, sequencing methods for older material are advancing [19] in tandem with the recognition that these collections represent an unparalleled resource [20].

Another near-term goal for the reference library is to assess additional ground beetle subfamilies. To date, only six of 15 subfamilies have been sampled and although CO1 is a generally successful marker (this work, [21]), there are known issues for identifying particular groups (e.g. *Bembidion*, [22], *Cicindela* [23]). The other common issues with this marker, including incomplete lineage sorting and introgression (or hybridization) have not appeared to hinder our efforts in a significant way thus far. Heteroplasmy (multiple mitochondrial haplotypes within a single individual) is not common in Metazoa [24] and we have not found this to be an issue in our samples. Nuclear copies of mitochondrial DNA (numts) have been identified in less than 1% of NEON sequences to date and can easily be screened by examining trace files for multiple peaks and translation to the amino acid sequence. Symbiont-induced selective sweeps, which can cause linkage disequilibrium with mitochondrial DNA, have been found in mosquitoes [25], and there is the potential for this to affect ground beetles [26]. Additional records from wider geographic ranges to obtain species’ full genetic diversity, and additional genes (e.g. 28S for ground beetles, more quickly evolving genes for species such as *Ae. fitchii*) will aid in resolving issues where they occur. Ideally gene trees for multiple unlinked genes [27] or the use of amplified fragment length polymorphisms [28] could be included to clarify problematic groups. The identification issues outlined above (sequenced specimens being discordant) as well as the potential hurdles outlined here could both be ameliorated with more data from additional specimens (i.e. expertly identified material collected by NEON or other research campaigns, and properly determined museum specimens). For these reasons, it is important that users of NEON data integrate with the observatory to ensure the efficacious build-out of these resources.

In the construction of the integrated reference library NEON will continue to consult with taxonomic experts and finalize checklists (which will control the entry of information by reconciling common misspellings or outdated taxonomy) of Linnaean species names for ground beetles and mosquitoes in the NEON purview. These names are properly published and recognized by the International Commission of Zoological Nomenclature, and associated references will also be cited (e.g. pertinent species descriptions and revisions) and experts will have many opportunities to comment on and edit these lists. Further, NEON is working to ensure that appropriate database fields are being included to accommodate particular kinds of information that are not standardly available (e.g., subgenera for mosquitoes, and sampling and preservation methods) as well as the possibility to readily accommodate new technologies as they become widely used for identification, e.g. near infrared spectroscopy for metabolomics [29]. Ideally, this web-based, pro-amateur and expert-sourced reference library could act as a clearinghouse for new behavioral, ecological or distribution information, newly realized morphological or molecular characters, or changes in phenology, similar to scratchpads (http://scratchpads.eu/), and akin to the vision by the previously-funded NSF Planetary Biodiversity Inventories.

The successful outcome from our evaluation of novice parataxonomists is similar to other researchers’ work, where parataxonomists form a critical part of the workflow for large, successful, biodiversity inventories [30], [31]. Therefore, a subset of collected specimens will serve as synoptic teaching collections for parataxonomists at each of the 20 NEON Domain support facility laboratories. This will aid in parataxonomists’ ability to sort specimens to morphospecies and in turn, ease the burden on experts conducting NEON’s morphological identifications. The vast majority of specimens collected (including the bycatch), however, will be archived in a distributed set of collections (the plan for which is undergoing additional development during NEON’s construction period).

Data from this integrated system for ground beetles and mosquitoes have already been fruitful. For instance, although the NEON sub-sampled mosquito specimen, *Psorophora discolor* culicid2273 (Ps-79), from the Walter Reed Biosystematics Unit (WRBU; likely collected in the 1960 s or 70 s by L.E. Rozeboom, in Oklahoma) amplified only a partial read of 307 bp, these data verified a range expansion of this species. From our most recent collections in 2011 (data are in their final verification steps), we found *P. discolor* at CPER, CO. Although there were no other valid records for this species from BOLD or GenBank, we found a 305 of 307 bp (99%) match to our sequence in ‘NEONZ’. The westernmost distribution of this species was previously known from Oklahoma at 40.8**°** latitude. In addition, unknown specimens may receive species level determinations from other specimens that have been identified and then sequenced during museum visits. On our initial museum visit (University of Colorado Museum of Natural History, UCM), several ground beetle specimens were sub-sampled from material collected in 2001 and 2002. These specimens were carabid662 (UCM 0070970), carabid664 (UCM 0070972), and carabid666 (UCM 0070974) and resulted in barcode compliant sequences, however they were retained in the ‘NEONZ’ project as they were identified only to the genus level. On a subsequent visit to C.P. Gillette Museum of Natural History at Colorado State Univ. (CSUC) we generated sufficient sequence data such that these specimens matched *Cicindela punctulata* 100%, *C. obsoleta* 99.7%, and *C. tranquebarica* 99.4%, respectively. Given that these specimens can now be assigned with species attributions using molecular data, this information should be captured both on the determination label on the pinned specimen (e.g. det. DNA: CO1, 2011) and as an additional standardized field in databases (basis of identification  =  morphological, molecular, etc.). It is also for these reasons that the ‘NEONZ’ project is public and able to be integrated with data generated by others and with our own ongoing work.

Once completed, NEON’s integrated terrestrial insect identification reference library will serve as a bridge for a variety of users during operations to access up-to-date ecological and evolutionary research findings. For instance, ecologists will be able to use NEON’s tools and specimens as a resource for comprehensive taxonomic information and for understanding changes in populations over time and in response to varying land use types, e.g. through isotopic analyses of NEON’s archived specimens [32]. Understanding trophic relationships, e.g. gut contents in ground beetles [33], and host specificity, e.g. bloodmeals in mosquitoes [34] will be possible by sequencing the abdominal contents of these specimens. Ground beetle species’ invasions or geographic shifts will be made more straightforward by the extensive DNA barcode reference library [20], [35]. Citizen scientists will be able to learn about species’ contributions to particular ecosystem services, and generate site-specific information sheets, including many high-quality images that represent the possible variation in species of interest. Epidemiologists will be able to monitor juvenile mosquitoes slated for collection by NEON’s Aquatic observing platform. This will provide an unprecedented opportunity to understand larval mosquito ecology and for the prediction of adult distributions of these important disease vectors and their subsequent connection to human cases. Conservation biologists can assess distribution of the phenology of rare or threatened species, and track indicator species in relation to changing habitats, such as areas with heavy insecticide use or increasing urbanization. Further, we anticipate that the documented construction of this resource can serve as a reference for others building similar ventures globally, e.g. the Group on Earth Observations Biodiversity Observation Network (GEOBON), the South African Environmental Observation Network (SAEON), Australia’s Terrestrial Ecosystem Research Network (TERN), and the European Biodiversity Observation Network (EBONE).
